# Supplementary figures and images for: A fine-tuned vector-parasite dialogue in tsetse's cardia determines peritrophic matrix integrity and trypanosome transmission success
Source: PLoS Pathog. 2018 Apr 3;14(4):e1006972. doi: 10.1371/journal.ppat.1006972 (PMC5898766; doi:10.1371/journal.ppat.1006972)

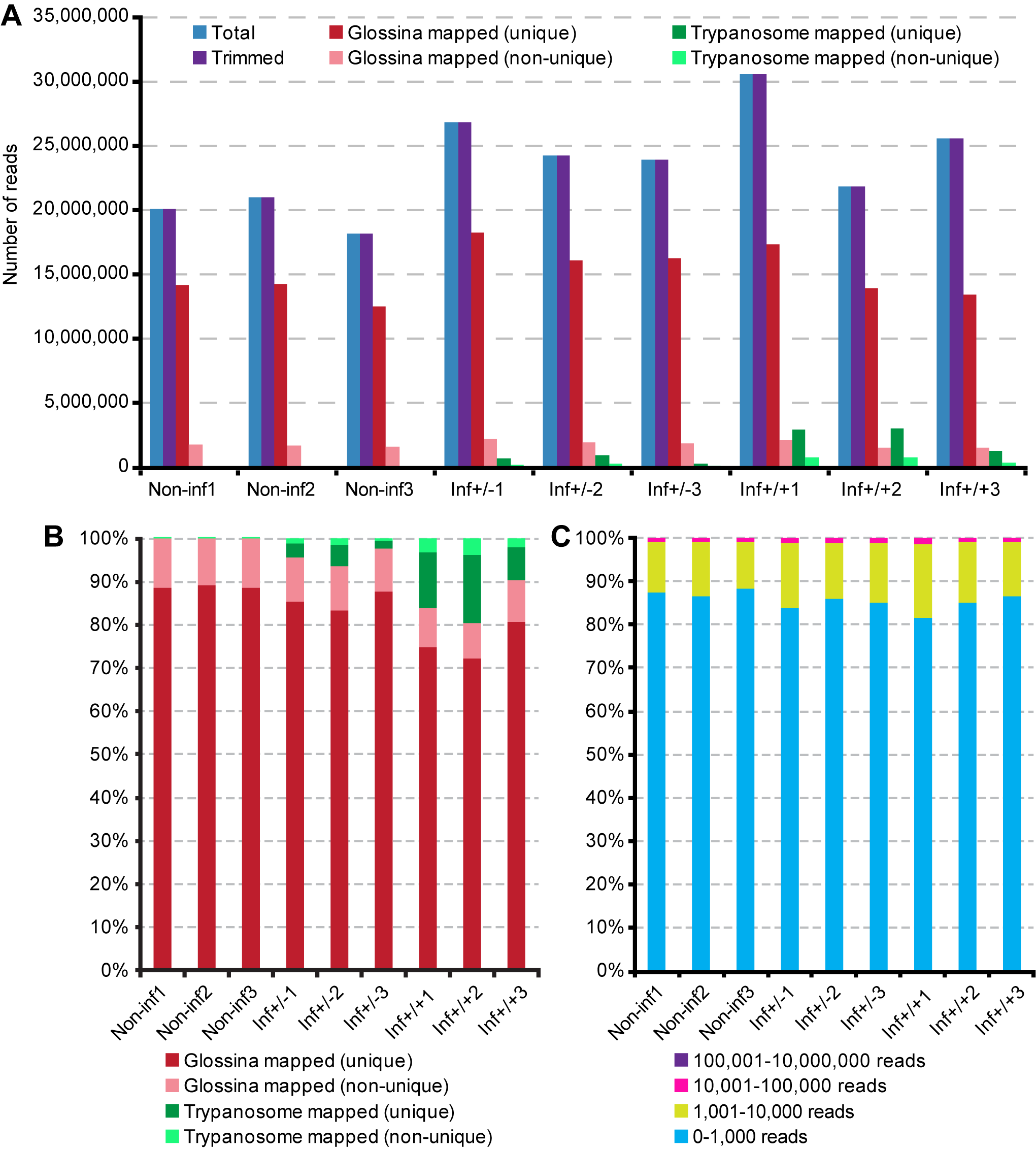

Supplement: S1 Fig — (A) Number of RNA-seq reads in each of three biological replicates from Non-inf, Inf +/- and Inf +/+ cardia. (B) Proportion of total trimmed reads that map to Glossina morsitans morsitans or Trypanosoma brucei brucei 927. (C) Percent relative abundance of mapped Glossina morsitans morsitans transcripts. (TIF) [file ppat.1006972.s001.tif]

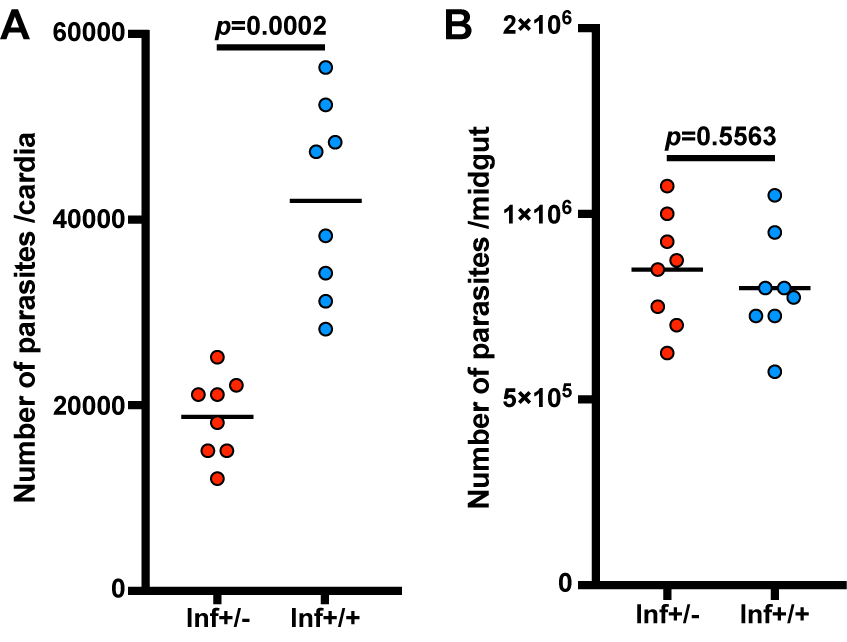

Supplement: S2 Fig — (A) Number of parasites in the cardia of Inf+/- (red) and Inf+/+ (blue) flies. (B) Number of parasites in the midgut of Inf+/- (red) and Inf+/+ (blue) flies. The black bar represents the mean of the replicates for each treatment. Midgut and cardia were dissected from eight 40 days-old females. Parasites were counted using a hemocytometer. Statistical analyses were carried out using the non-parametrical Mann-Whitney rank test. (TIF) [file ppat.1006972.s002.tif]

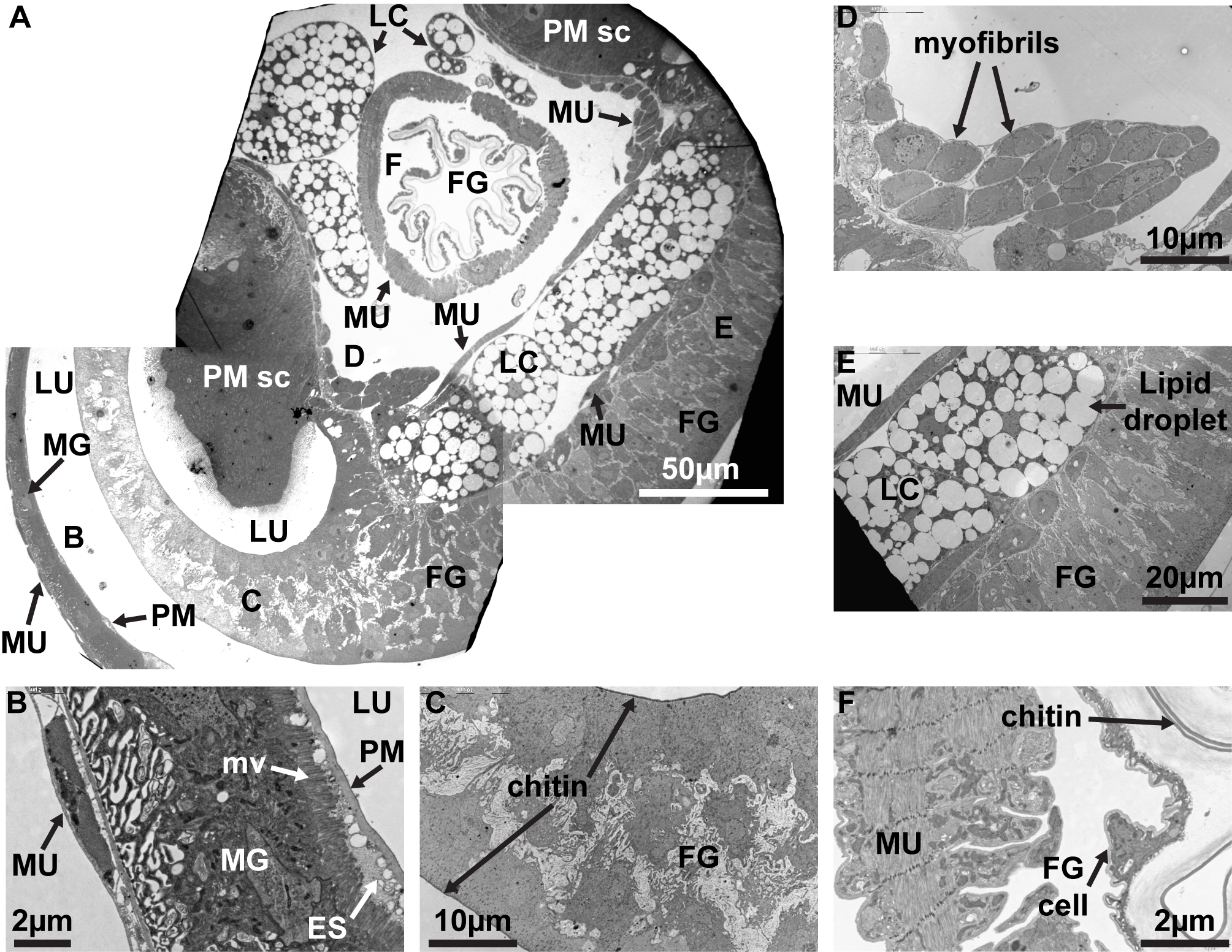

Supplement: S3 Fig — (A) Transversal section of a non-infected cardia. Two pictures of the same cardia were merged to produce a larger picture. B, C, D, E and F are magnified micrographs of cardia tissues. (B) Midgut tissue delimiting the outer part of the cardia. (C) Foregut tissue invagination within cardia, corresponding to the stomodeal valve in other insects. (D) Myofibrils assembled to form the sphincter surrounding the foregut opening in the cardia. (E) Lipid-containing cells, immediately adjacent to foregut tissue, covered by a thin layer of muscle. (F) Foregut tube coming out of the cardia. LU: Lumen; MG: Midgut; PM sc: PM secreting cells; FG: Foregut; MU: muscle; LC: Lipid-containing cells; mv: microvilli; ES: Ecotperitrophic space. (TIF) [file ppat.1006972.s003.tif]

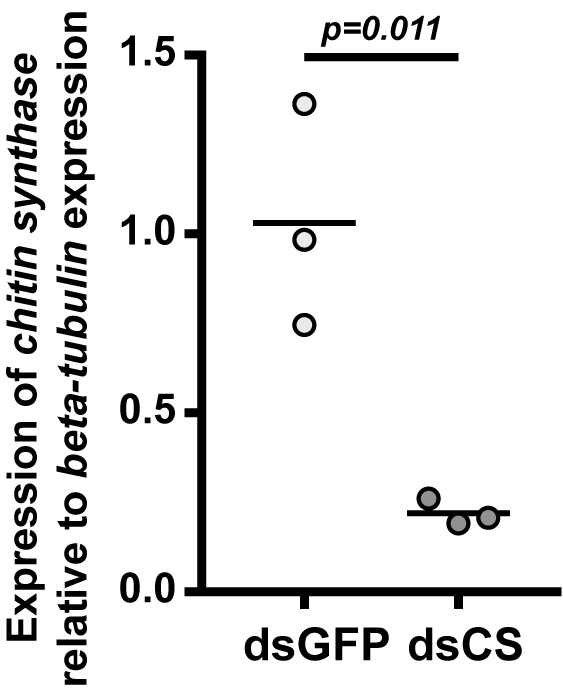

Supplement: S4 Fig — Expression of chitin synthase relative to constitutively expressed ß-tubulin after treatment with dsRNA-gfp (control; white circles) and dsRNA-chitin synthase (dsCS; gray circles). chitin synthase expression is significantly decreased after RNAi knockdown (Student t-test, p = 0.011). (TIF) [file ppat.1006972.s004.tif]

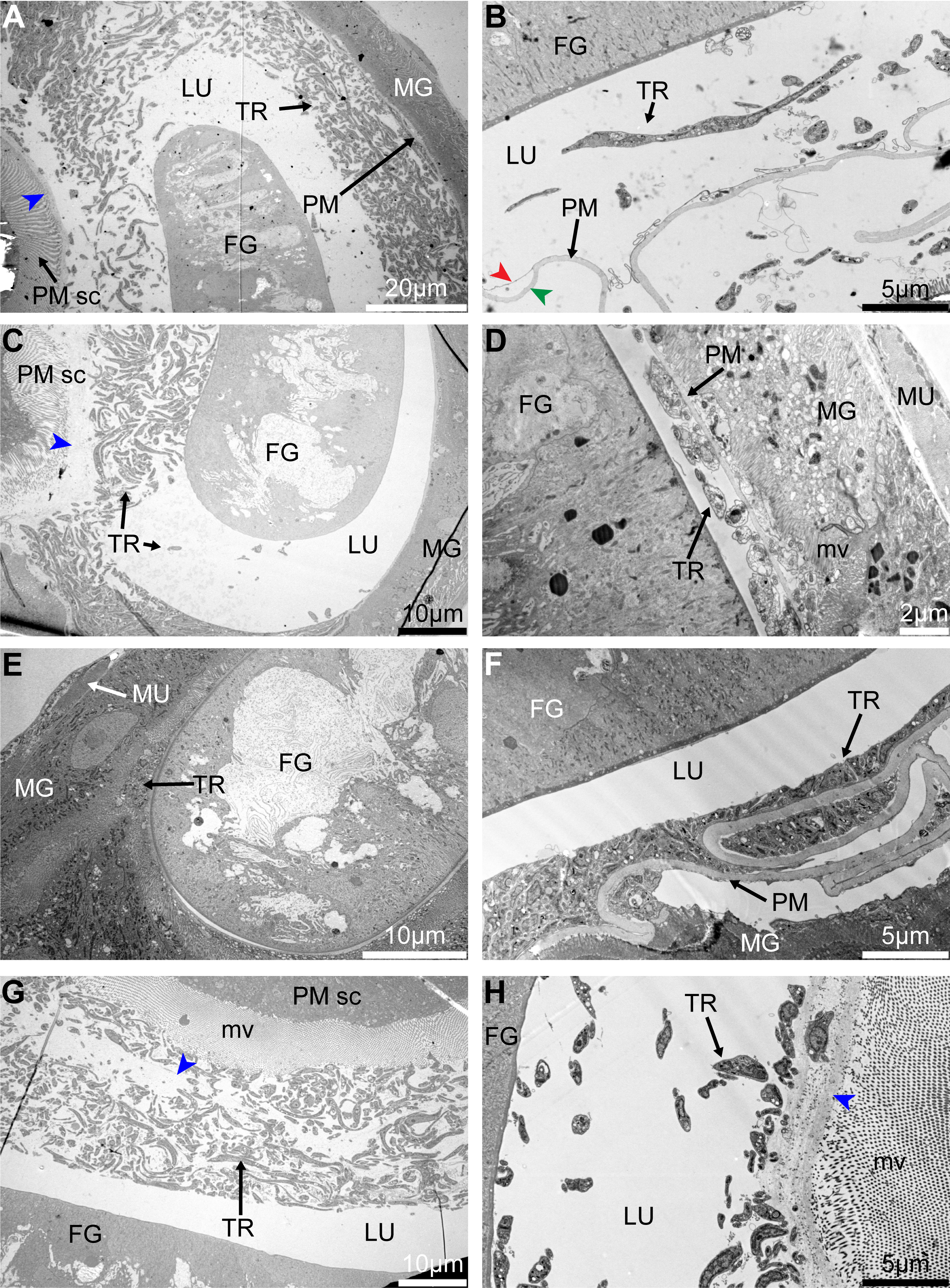

Supplement: S5 Fig — Right panels present accumulation of parasites next to the PM secreting cells in the cardia. Left panels present trypanosomes next to mature PM. Micrographs in this image represent four of six biological replicates. Right and left panels are paired to correspond to a same individual. Blue arrowheads: newly secreted PM; red arrowhead: PM electron-dense layer; green arrowhead: PM electron-lucent layer; MG: midgut; PM sc: PM secreting cells; mv: microvilli; TR: trypanosomes; LU: lumen; FG: foregut; MU: muscle. (TIF) [file ppat.1006972.s005.tif]

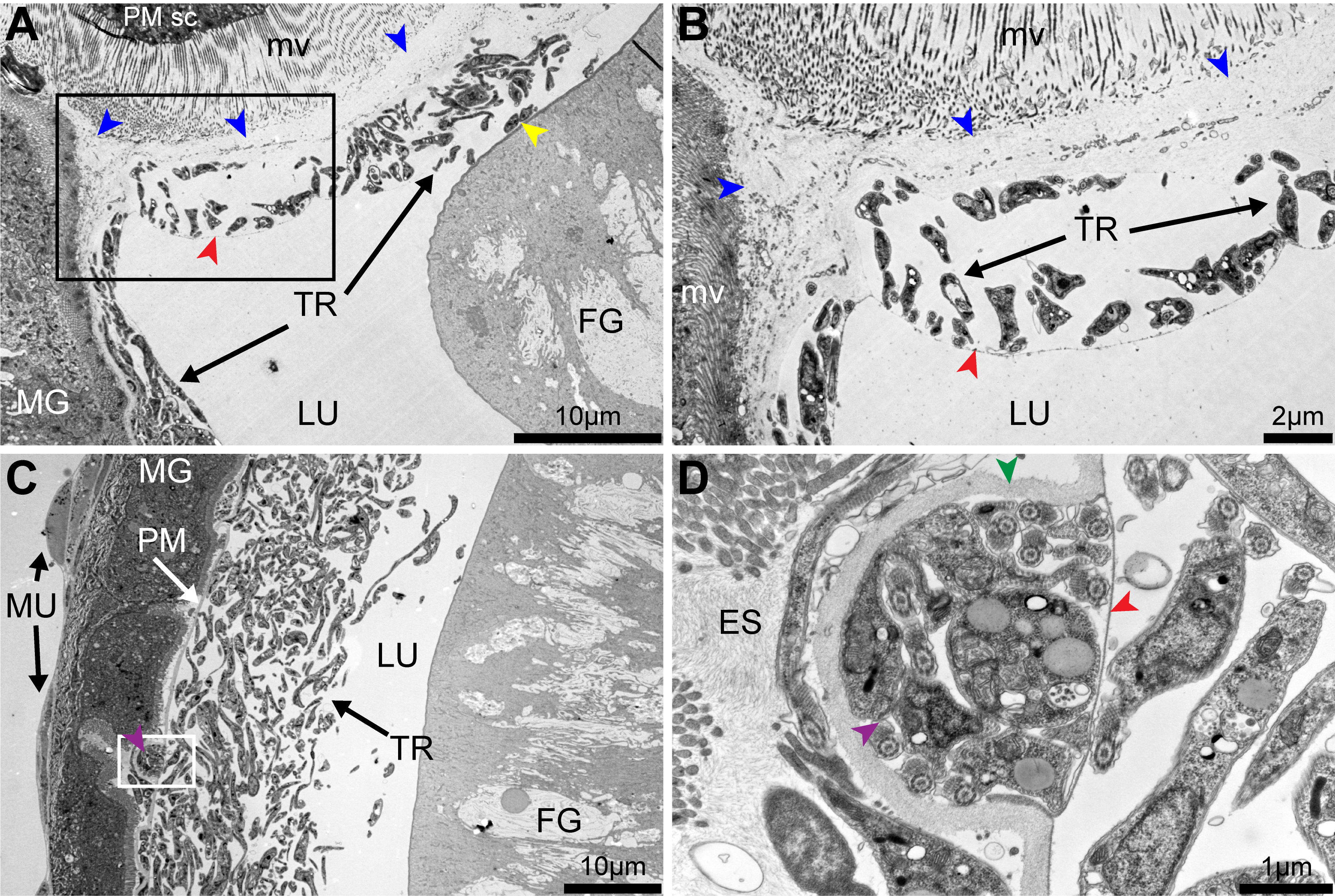

Supplement: S6 Fig — (A) Ultrastructure of the cardia in the region where the PM is secreted. Trypanosomes are observed either trapped inbetween the electron-dense layer of the PM (red arrowhead) and newly synthesized PM secretions (blue arrowheads), or free in the lumen and close to the foregut (yellow arrowhead). (B) is a magnified micrograph of the black frame in (A). (C) Ultrastructure of the region below the annular cleft where the PM is secreted. Trypanosomes are observed in the ES, in the lumen and trapped in the PM as cyst-like bodies (purple arrowheads). (D) Magnified micrograph of the white frame in (A). A cyst-like body (purple arrowhead) is entrapped inbetween the electron-dense (red arrowhead) and electron-lucent layers of the PM (green arrowhead). The ultrastructure presented in micrographs (A) and (C-D) originated from 2 different inf+/+ cardia. Micrographs in this image represent 2 two of six of biological replicates from cardia inf+/+. MG: midgut; PM sc: PM secreting cells; mv: microvilli; TR: trypanosomes; LU: lumen; FG: foregut; MU: muscle; ES: ectoperitrophic space. (TIF) [file ppat.1006972.s006.tif]

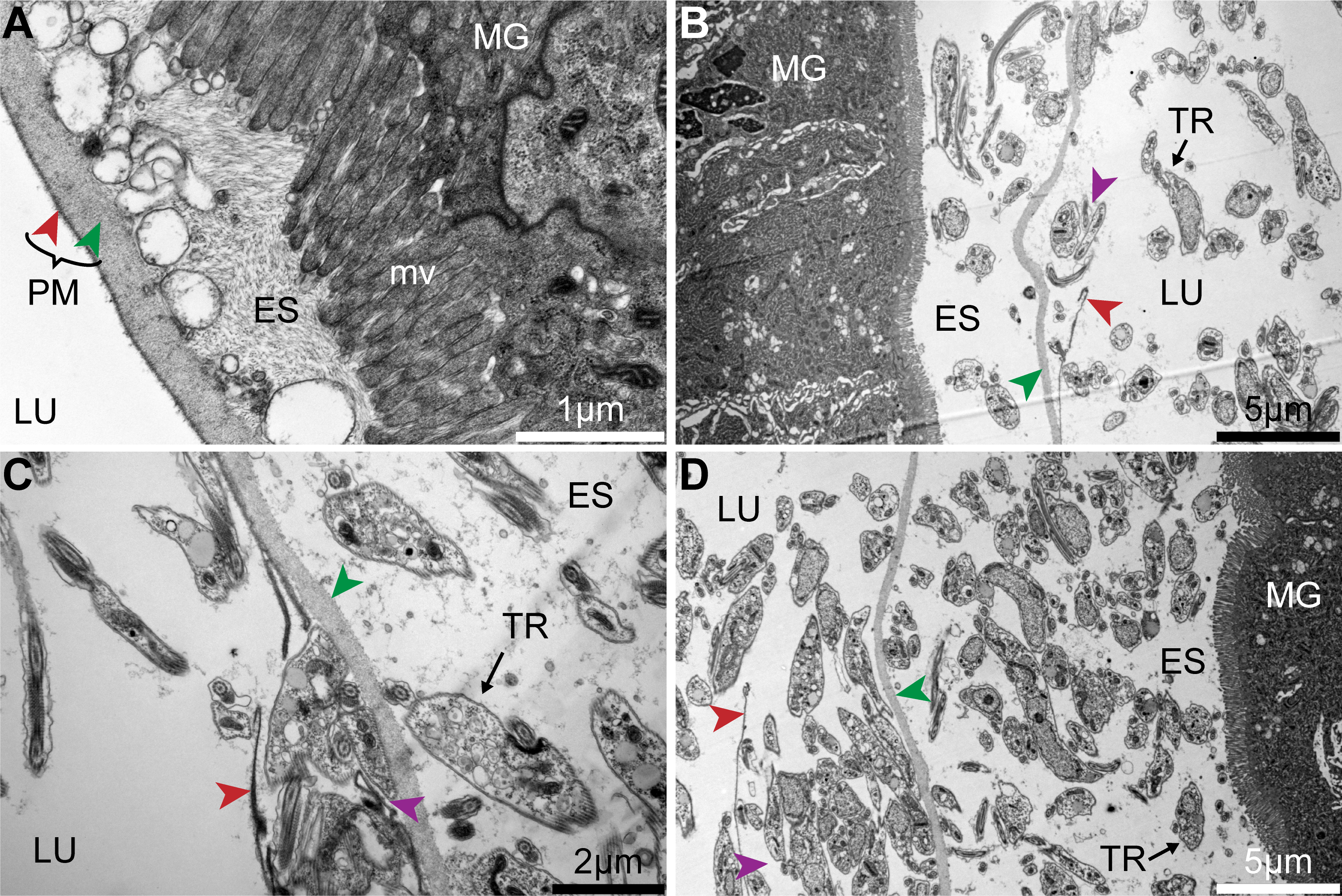

Supplement: S7 Fig — (A) PM ultrastructure in cardia of non-inf tsetse. The electron-dense (red arrowhead) and electron-lucent (green arrowhead) layers of a mature PM are intact in these flies (B-D) Close-up images of disrupted electron-dense layer of the PMs. Cyst-like bodies (purple arrowheads) are observed inbetween the two layers of the PM. Micrographs in this image represent one and three of three and six of biological replicates from cardia non-inf and inf+/+, respectively. PM: peritrophic matrix; MG: midgut; mv: microvilli; TR: trypanosomes; LU: lumen; ES: ectoperitrophic space. (TIF) [file ppat.1006972.s007.tif]

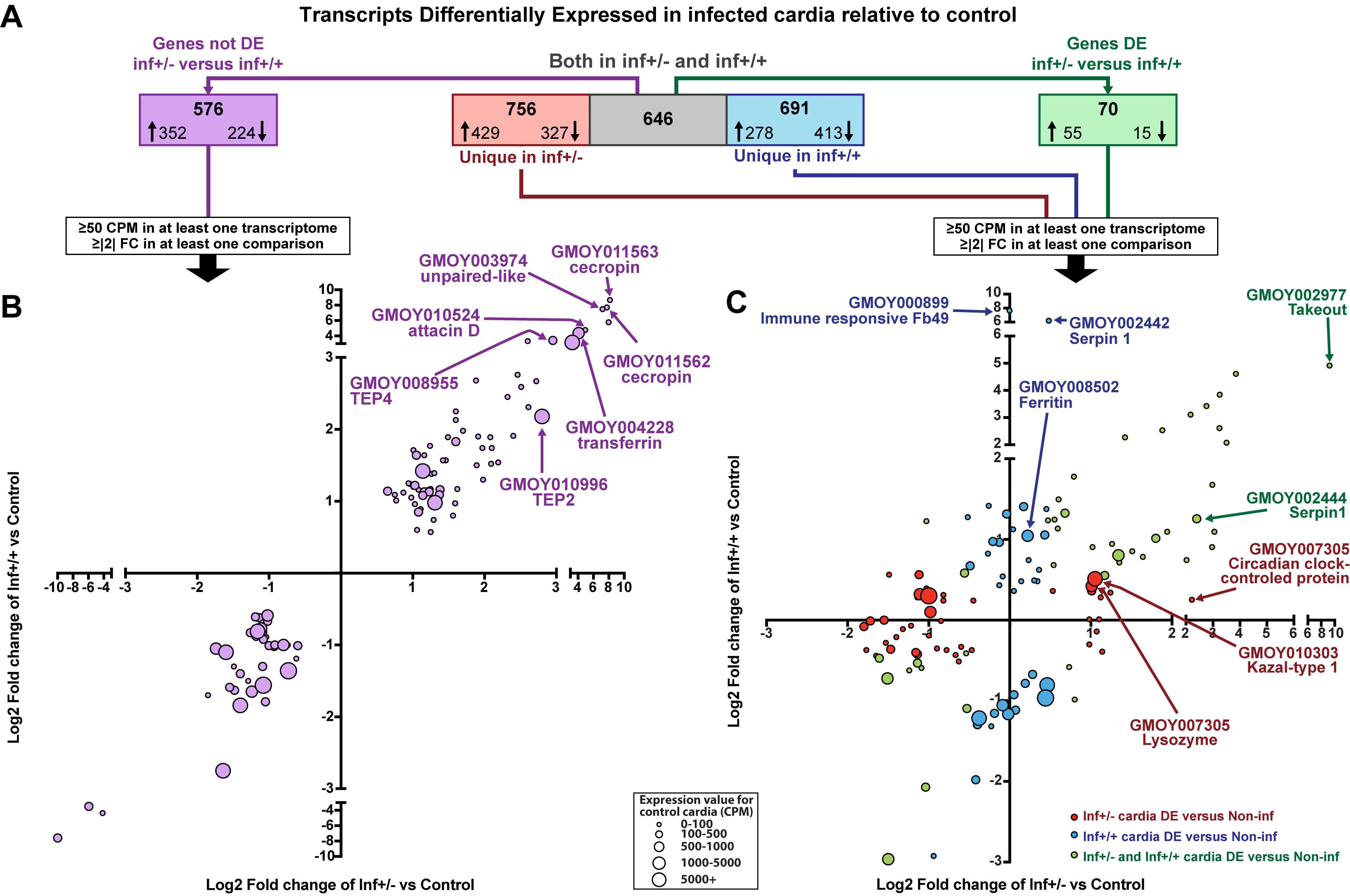

Supplement: S8 Fig — (A) A total of 2,093 transcripts were DE in inf+/- and inf+/+ cardia relative to uninfected (non-inf) controls. In inf+/- cardia (red), 429 and 327 transcripts were up and downregulated, respectively. In inf+/+ cardia (blue), 278 and 413 transcripts were up and downregulated, respectively. Of the DE transcripts shared by both infection phenotypes (646), 576 are similarly regulated, while 70 show DE in inf+/- versus inf+/+ phenotypes. (B-C) Transcripts are plotted as a function of their fold-changes (Log2 scale) obtained by comparison between control non-inf transcriptome and either inf+/+ (y-axis) or inf+/- (x-axis) transcriptome. The size of the circle indicates the expression value (CPM) in the control non-inf cardia. The genes presented have been annotated with their genome ID number and their best BLASTx annotation. Panel B shows similarly regulated transcripts in both cardia infection phenotypes. Panel C shows differentially regulated transcripts between the two cardia infection phenotypes. In panel C, DE transcripts expressed exclusively in inf+/+ compared to non-inf are shown in blue, DE transcripts expressed exclusively in inf+/- compared to non-inf are shown in red, and DE transcripts expressed in both inf+/+ and inf+/- compared to non-inf are shown in green. (TIF) [file ppat.1006972.s008.tif]

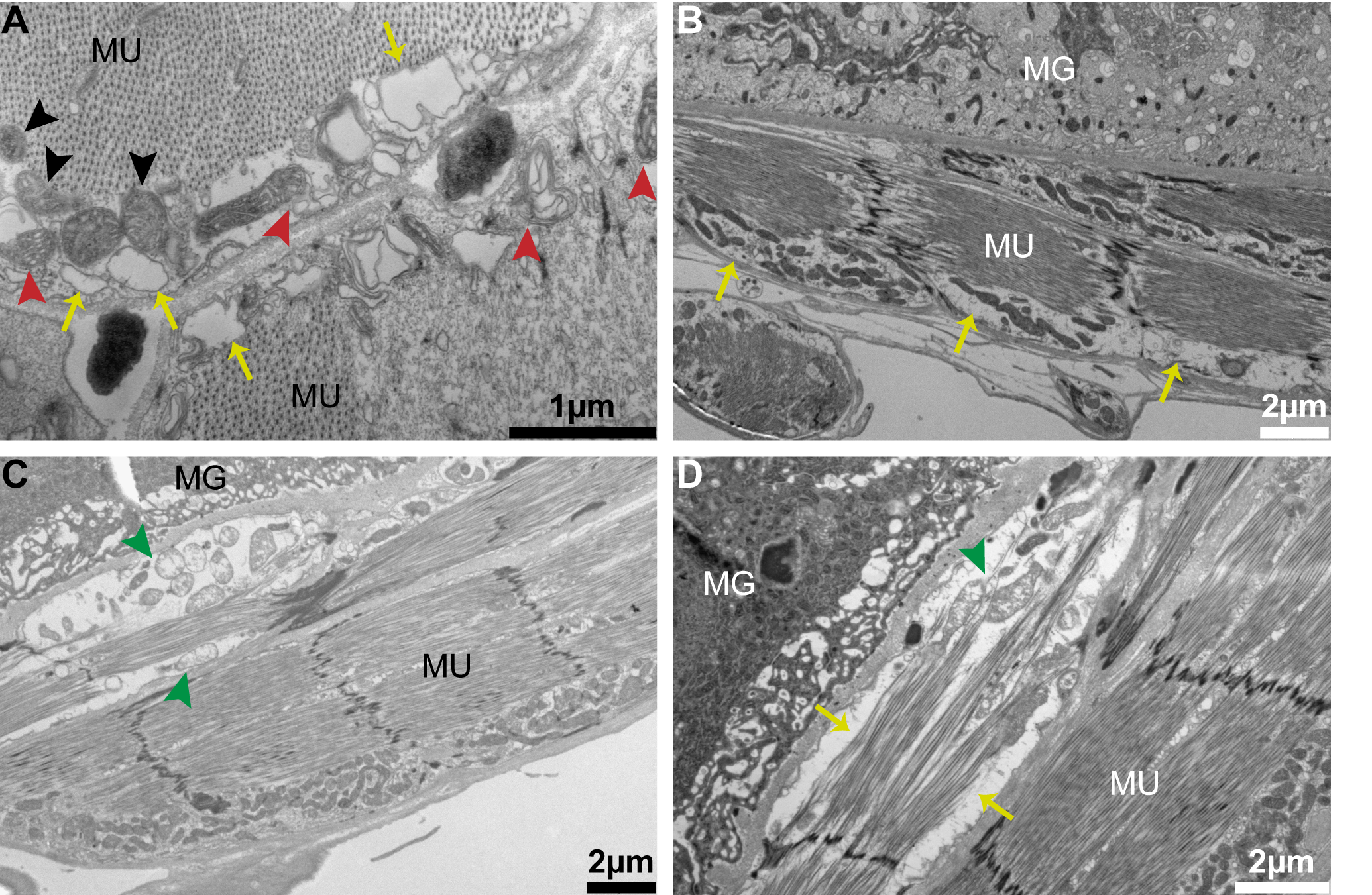

Supplement: S9 Fig — (A) Transversal section of muscle tissues (MU) composing the sphincter. (B-D) Longitudinal section of muscles (MU) layering the midgut tissues. Micrographs in this image represent three of five of biological replicates from cardia inf+/-. Black arrowheads: healthy mitochondria; red arrowheads: vacuolation of mitochondria; yellow arrow: sarcoplasmic dilatation; green arrowheads: swelling mitochondria; MG: midgut. (TIF) [file ppat.1006972.s009.tif]
